# Sediment Burial Intolerance of Marine Macroinvertebrates

**DOI:** 10.1371/journal.pone.0149114

**Published:** 2016-02-22

**Authors:** Vicki J. Hendrick, Zoë L. Hutchison, Kim S. Last

**Affiliations:** Scottish Association for Marine Science (SAMS), Oban, PA37 1QA, United Kingdom; Bangor University, UNITED KINGDOM

## Abstract

The marine environment contains suspended particulate matter which originates from natural and anthropogenic sources. Settlement of this material can leave benthic organisms susceptible to smothering, especially if burial is sudden i.e. following storms or activities such as dredging. Their survival will depend on their tolerance to, and their ability to escape from burial. Here we present data from a multi-factorial experiment measuring burial responses incorporating duration, sediment fraction and depth. Six macroinvertebrates commonly found in sediment rich environments were selected for their commercial and/or conservation importance. Assessments revealed that the brittle star (*Ophiura ophiura*), the queen scallop (*Aequipecten opercularis*) and the sea squirt (*Ciona intestinalis*) were all highly intolerant to burial whilst the green urchin (*Psammichinus miliaris*) and the anemone (*Sagartiogeton laceratus*), showed intermediate and low intolerance respectively, to burial. The least intolerant, with very high survival was the Ross worm (*Sabellaria spinulosa*). With the exception of *C*. *intestinalis*, increasing duration and depth of burial with finer sediment fractions resulted in increased mortality for all species assessed. For *C*. *intestinalis* depth of burial and sediment fraction were found to be inconsequential since there was complete mortality of all specimens buried for more than one day. When burial emergence was assessed *O*. *ophiura* emerged most frequently, followed by *P*. *miliaris*. The former emerged most frequently from the medium and fine sediments whereas *P*. *miliaris* emerged more frequently from coarse sediment. Both *A*. *opercularis* and *S*. *laceratus* showed similar emergence responses over time, with *A*. *opercularis* emerging more frequently under coarse sediments. The frequency of emergence of *S*. *laceratus* increased with progressively finer sediment and *C*. *intestinalis* did not emerge from burial irrespective of sediment fraction or depth. Finally, and perhaps unsurprisingly, the greatest ability to emerge from burial in all other species was from shallow (2 cm) burial. Although survival was consistently highly dependent on duration and depth of burial as expected, emergence behaviour was not as easily predictable thereby confounding predictions. We conclude that responses to burial are highly species specific and therefore tolerance generalisations are likely to be oversimplifications. These data may be used to inform environmental impact models that allow forecasting of the cumulative impacts of seabed disturbance and may provide mitigation measures for the sustainable use of the seabed.

## Introduction

The marine environment contains particulate matter, both organic and inorganic in origin, some of which accumulates on the seabed whilst some is suspended in the water column and termed suspended particulate matter (SPM). Near-seabed turbulence from waves, tides, currents and freshwater run-off typically results in sediment re-suspension, mobilisation and eventual deposition [1] leading to elevated SPM levels, particularly in near shore habitats. For this reason, the sediment-water interface in shallow coastal water is often highly dynamic with erosion, transport, and deposition varying in magnitude over different time scales [2].

Offshore anthropogenic activity is also typically concentrated in coastal waters and many industries contribute to sediment disturbance, for example estuarine and aggregate dredging [3], cable trenching and pile driving [4], the burgeoning marine renewable industry [5–8] and the extensive fortification and embankment of coasts [9]. Whilst there is general consensus that large scale change in sediment dynamics are anticipated with increasing use of the coasts and seabed, it is hard to predict the environmental consequences of such activities. An increase in meteorological extremes including storm frequency that is predicted due to climate change [10, 11] is also likely to affect sediment dynamics with associated increases in coastal SPM.

The importance of SPM to marine organisms, and their sensitivity to changes in SPM levels, is dependent on the behaviour of those species and their adaptation to a particular level of SPM or depositional regime [12]. This is of particular relevance to those organisms living on the seabed. Filter, deposit and suspension feeders for example, many of which are largely sedentary, often require suspended and/or deposited material to supplement their diets, for camouflage or as building material for dwelling tubes as is the case with Sabellarid polychaetes [13, 14]. Conversely, large increases in deposited particulate matter above natural ranges, due to dredging activities for example, may be such that sedentary or slow moving organisms are at risk of smothering if they are unable to survive burial or escape/emerge from burial [15, 16].

Smothering of habitats and species assemblages leading to shifts in community structure, composition and faunal abundance, is well documented [17]. Impacts are usually physical i.e. direct smothering, and/or chemical due to toxicity from resuspended contaminated sediments (RCS) as often occurs from industrial port dredging activities [18, 19]. Community structures will change not only at the site of impact but often much further afield, sometimes evident kilometres from the site of origin [18]. Benthic recovery to a pre-disturbance level, can be fast (within months) in fine grained deposits dominated by opportunistic species [20, 21], though has also been shown to take years, if at all [22].

Although changes in community structure are well documented following such sedimentary disturbance, there is limited data on species-specific behaviours and intolerances [23]. An exception is an early study of 25 species of bivalves, which showed that epibenthic suspension-feeders which used byssal attachment were more susceptible to smothering, albeit from natural causes, than those which did not, as evidenced by their presence in the fossil record [24]. The importance of mobility to burial intolerance, and of sediment type, has also been demonstrated in a range of invertebrates as important considerations in burial survival [12, 25].

To address the paucity of burial intolerance data we conducted experimental burial events in laboratory aquaria to compare the responses of eight common macroinvertebrate species: the brittle star (*Ophiura ophiura*), Ross worm (*Sabellaria spinulosa*), Queen scallop (*Aequipectin opercularis*), green urchin (*Psammechinus miliaris*), yellow sea squirt (*Ciona intestinalis*) and an anemone (*Sagartiogeton laceratus*). Furthermore, for the purposes of discussion we include comparative assessments from another study that has used the same burial protocol as described here, for the blue mussel (*Mytilus edulis*) and horse mussel (*Modiolus modiolus*) [26] to which readers are referred. The choice of species was based on: a) a wide UK distribution including the English Channel and Southern North Sea where activities such as aggregate dredging and renewable energy developments etc. are common; b) differing seabed type preferences; c) the diverse range of behavioural and physiological traits; and d) their conservation and/or commercial importance. Whilst some theoretical assessment of intolerance to burial have been made in some of these species, inferred from the literature and summarised as part of the MarLIN sensitivity assessment [23], there is a general lack of empirical data upon which to make informed management decisions, as is required for regulatory licencing of many seabed activities.

A multifactorial experimental design was used to test burial intolerance using a range of sediment fractions for various durations and under different burial depths all of which have previously been shown to impact survival and/or emergence in benthic invertebrates [12, 27, 28].

## Methods and Materials

### Specimen Collection

Specimen collection for this experimental study was as follows: *Ophiura ophiura*, *Psammichinus miliaris*, *Ciona intestinalis* and *Sagartiogeton laceratus* and specimens were collected locally around Dunstaffnage, Argyll (55° 27’ 17.65”N, 05° 26’ 0.18”W; WGS 84 datum), by divers from the National Facility of Scientific Diving facility (NFSD) at SAMS. No special permissions for these collections were required and numbers were kept to the minimum necessary to satisfy robust statistical comparisons. *Sabellaria spinulosa* specimens were collected off the Lincolnshire coast (53° 02’ 37.28”N, 00° 49’ 38.69”E; WGS 84 datum) by the Eastern Sea Fisheries Joint Committee and were not sourced from designated reef habitat. Cultured specimens of *Aequipecten opercularis* were sourced from a local producer Loch Fyne Seafarms Ltd. ensuring individuals were of a similar size and age.

All specimens were acclimatised in unfiltered seawater in flow-through stock tanks in the Alan Ansell Research Aquarium (at SAMS) for at least one week and in the experimental tanks for at least 36 hours prior to experimentation. All the experiments conducted complied with current laws regarding animal welfare in the UK and no permits were required for these experiments.

### Experimental Environment

All experiments were carried out in the research aquarium at SAMS during 2009 and 2010. Experiments took place in nine Paddle Vortex Resuspension Tanks (*p*VoRTs) a modification of those developed by Davies et al. [29]. The improved design incorporated an additional central paddle to generate current flow which reduced turbulence and increased flow consistency across the diameter of the tank. Paddle rotation was set at 16 rpm (± 0.5) providing a flow speed of 6–8 cm s^-1^ which facilitated oxygen exchange in the burial chambers, providing a more realistic protocol of natural conditions than a purely static set-up. All *p*VoRTs were supplied with a continuous flow of seawater (22 l h^-1^) sourced from sub-sand intakes in the Firth of Lorn, adjacent to SAMS laboratories. The nine *p*VoRTs and the specimen holding tanks were maintained under a 12 hour light—12 hour dark photoperiod including 15 minute dawn and dusk dimming periods simulating ambient photoperiods at the time of the experiments.

### Burial Protocol

Experimental specimens were measured, after which they were randomly allocated to treatments of burial in chambers. Chambers were capped PVC pipes with an internal diameter of 76 mm of suitable lengths to accommodate three different depths of sediment burial—shallow (2 cm), medium (5 cm) and deep (7 cm). The burial depths were chosen based on the expected increase in sediment levels within ≤ 500 m of the typical primary impact zone of marine aggregate dredging activity [21, 30]. Where possible additional specimens contained in chambers, but not buried, were included to control for the effects of burial, and further specimens without chambers were included to control for containment. The mobility of brittle stars and urchins precluded inclusion of free controls and their burial chambers required mesh covers to prevent escape. In addition the sea squirts had to be “tethered” with a weighted string to prevent them drifting out of the control (not buried) chambers. *S*. *spinulosa* specimens were isolated prior to burial as detailed in the next section.

Burial specimens were placed onto the base of the chambers and manually buried using kiln dried marine sediment. The burial depth of 2, 5 and 7 cm was measured from the dorsal surface of the animal. Sediments were obtained from a commercial supplier (Specialist Aggregates Ltd.) and three sediment fractions were used: coarse (described as 1.0–2.0 mm diameter), medium fine (size range verified by laser particle analysis using a Coulter LS230 and GRADISTAT software, as 0.25–0.95 mm) and fine (verified as 0.1–0.25 mm). The use of commercially obtained sediment provided homogeneity of the sediment fraction, lack of organic content and ready availability of large quantities.

The burials were replicated for durations of 1, 2, 4, 8, 16 and 32 days, or a variation thereof depending on burial intolerance. Thus the durations of burial were reduced for *A*. *opercularis* (0.5, 1.0, 1.5, 2, 4 and 16 days), *P*. *miliaris* (1, 2, 4, 6, 8, and 12 days) and the 32 day burial was omitted for *C*. *intestinalis*. The position of the chamber in *p*VoRT (inside/outside) and size of specimen were recorded as co-variables. Finally, following burial, the survival was determined using the behavioural indicators described in Table 1 over a range of durations.

**Table 1:** Indicators of mortality used to assess organisms following burial treatments.

Test Species	Behavioural trait	Mortality indicators	Duration of mortality assessment
*Ophiura ophiura*	Righting ability	Inability to right. Loss of spines.	60 minutes
*Sabellaria spinulosa*	Parapodial crawling	No crawling / movement even after palp contact with tweezers.	< 10 minutes
*Aequipecten opercularis*	Shell gape	Shell remains open even after mantle contact with tweezers.	< 1 minute
*Psammechinus miliaris*	Righting ability	Inability to right. Loss of spines.	60 minutes
*Ciona intestinalis*	Stem/trunk contraction	Inability to contract stem/trunk upon gentle squeezing.	48 hours
*Sagartiogeton laceratus*	Tentacle withdrawal reflex	Inability to retract or protract tentacles after mantle contact with tweezers or with food stimulation respectively.	48 hours

### Isolation of *Sabellaria spinulosa* dwelling tubes

**Fig 1:**
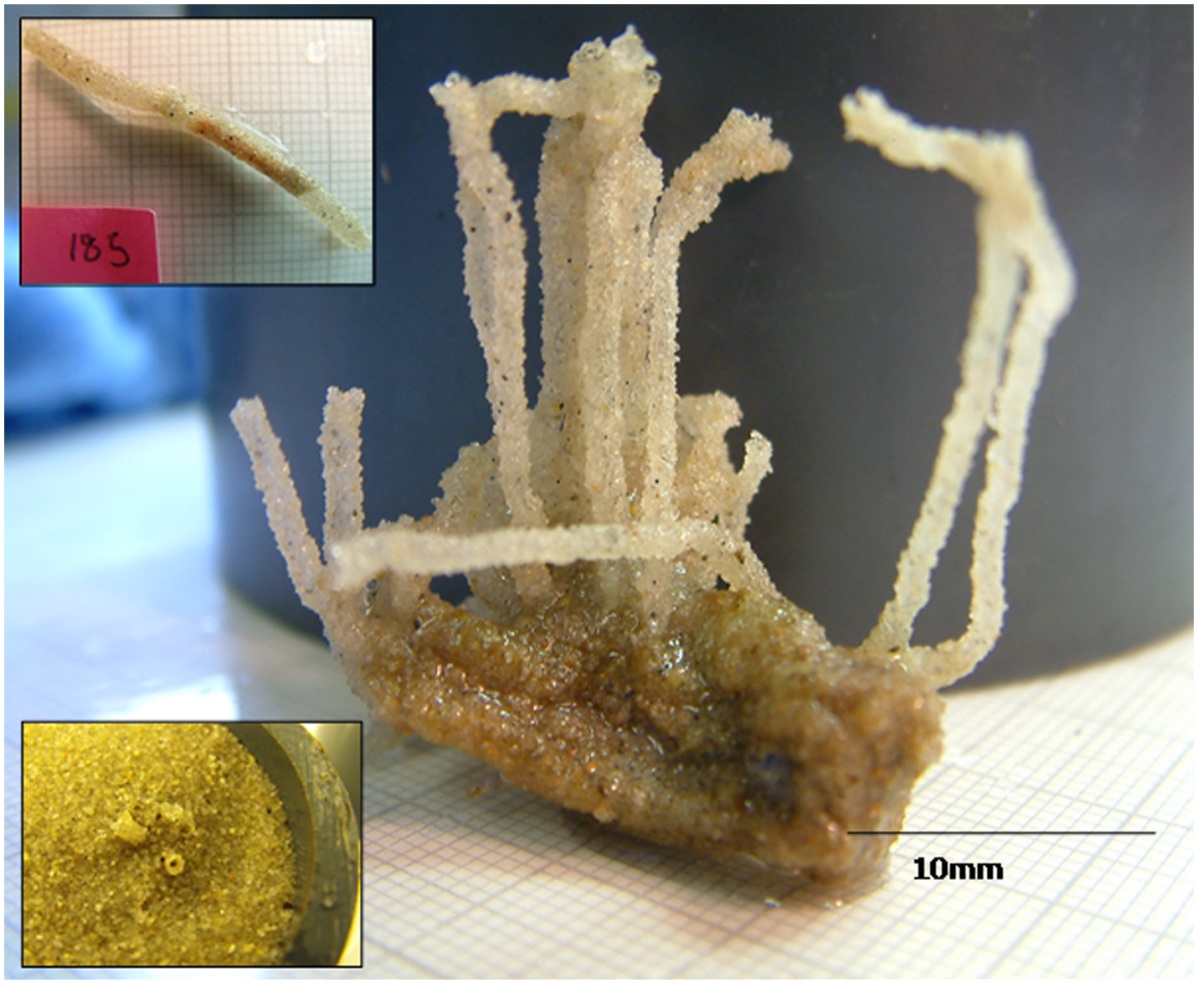
*Sabellaria spinulosa* clump with ‘emergence tubes’. ‘Emergence tubes’ constructed during burial by 2 cm fine (0.1–0.25 mm) sand for 16 days. The inset (top left) shows an isolated emergence tube which breaks off the main parent colony easily, and through which an individual animal is clearly visible. The inset (bottom left) shows three tubes emerging from the sediment following burial (Image source: Kim Last).

Assessment of the intolerance of *S*. *spinulosa* to burial events proved challenging due to the aggregation of their dwelling tubes when collected from the wild. There was also difficulty in assessing the physical condition of the polychaetes when retracted inside their tubes, necessitating isolation of individuals. To do this several small aggregated clumps were buried under the fine sediment for up to 32 days. This naturally induced the polychaetes to produce ‘burial emergence tubes’–a rapid elongation of dwelling tubes to the sediment-water interface (Fig 1) allowing easy detachment of individual tubes. Prior to experimental burial the inhabitant polychaetes were allowed to recover and repair any tube damage for two weeks. Intolerance to burial was then determined using the basic protocol described above, the only difference being that sediment from the burial chambers containing *S*. *spinulosa* tubes was sieved (1 mm) to recover the individual tubes following experimentation, due to their small size.

### Statistical approach

For all analysis of the response of each species, a binomial generalized linear model (GLM) with logit link was applied [31]. Firstly, the “drop1” function was used to justify the removal of the control data from subsequent analysis where a significant treatment affect was found. Maximal models incorporating all experimental variables (log_10_ of duration) and covariables, with and without appropriate interactions, were then systematically reduced using the drop1 function. This involved the removal of model terms above the significance threshold (set at *p* < 0.05), in a stepwise fashion. The model with the lowest Akaike Information Criterion (AIC) was considered to be the model which best described the experimental data. Simulated data were then used to plot the model, further confirming that the model was a good fit to the experimental data. The best fit minimal models and plots of the experimental data are reported in the next section.

## Results

In the following results, the treatment (burial) was a significant factor in the mortality of species as confirmed by the application of a binomial GLM. This provided justification for the removal of control data from subsequent analysis and is true for all species apart from *S*. *spinulosa*.

### Intolerance of *Ophiura ophiura* to burial

**Fig 2:**
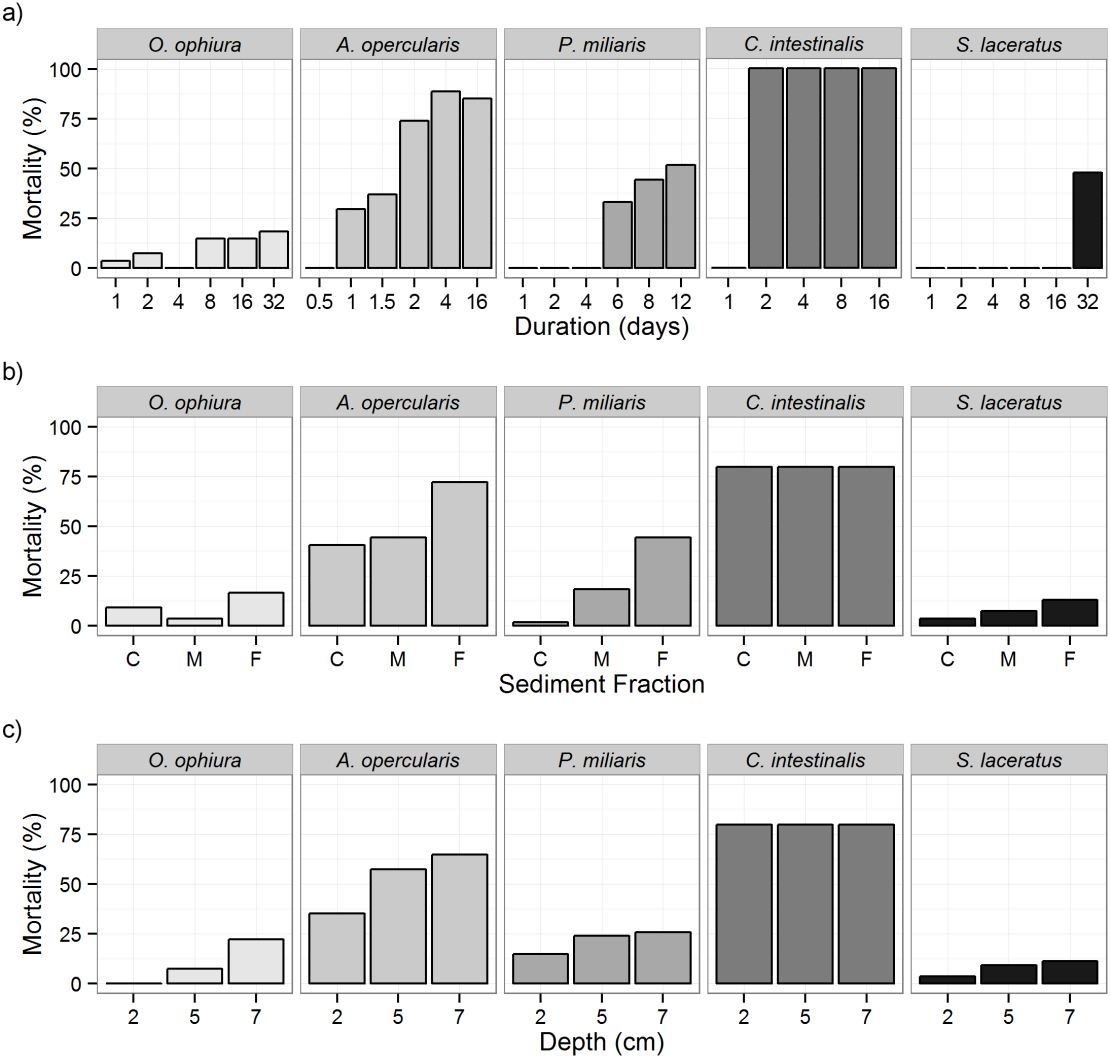
a-c. Multispecies assessment of mortality (%) in response to sediment burial. Factors tested: a) Duration (days); b) Sediment fraction (C = course, M = medium, F = fine) and; c) Depth of burial (cm) above organism.

The best fit minimal model for *O*. *ophiura* indicated that there was a significant interaction between the duration of burial and the sediment fraction, plus a second significant interaction between the depth of burial and the size of the individual (Table A in S1 File). Mortality increased with increasing duration of burial (max: 18.5% after 32 days, n = 27, Fig 2a) and was highest in the fine sediment fraction (16.7%, n = 54, Fig 2b) but lowest in the medium sediment (3.7%, n = 54). Mortality was highest in the deep burial (7 cm) where 22.2% of *O*. *ophiura* died (n = 54, Fig 2c). *O*. *ophiura* used in this study were between 3.9 cm and 10.1 cm ($\bar{\text{x}}$ = 6.7 cm, sd = 1.05), and higher mortality was found in larger *O*. *ophiura* ranging from 6 to 9.7 cm.

**Fig 3:**
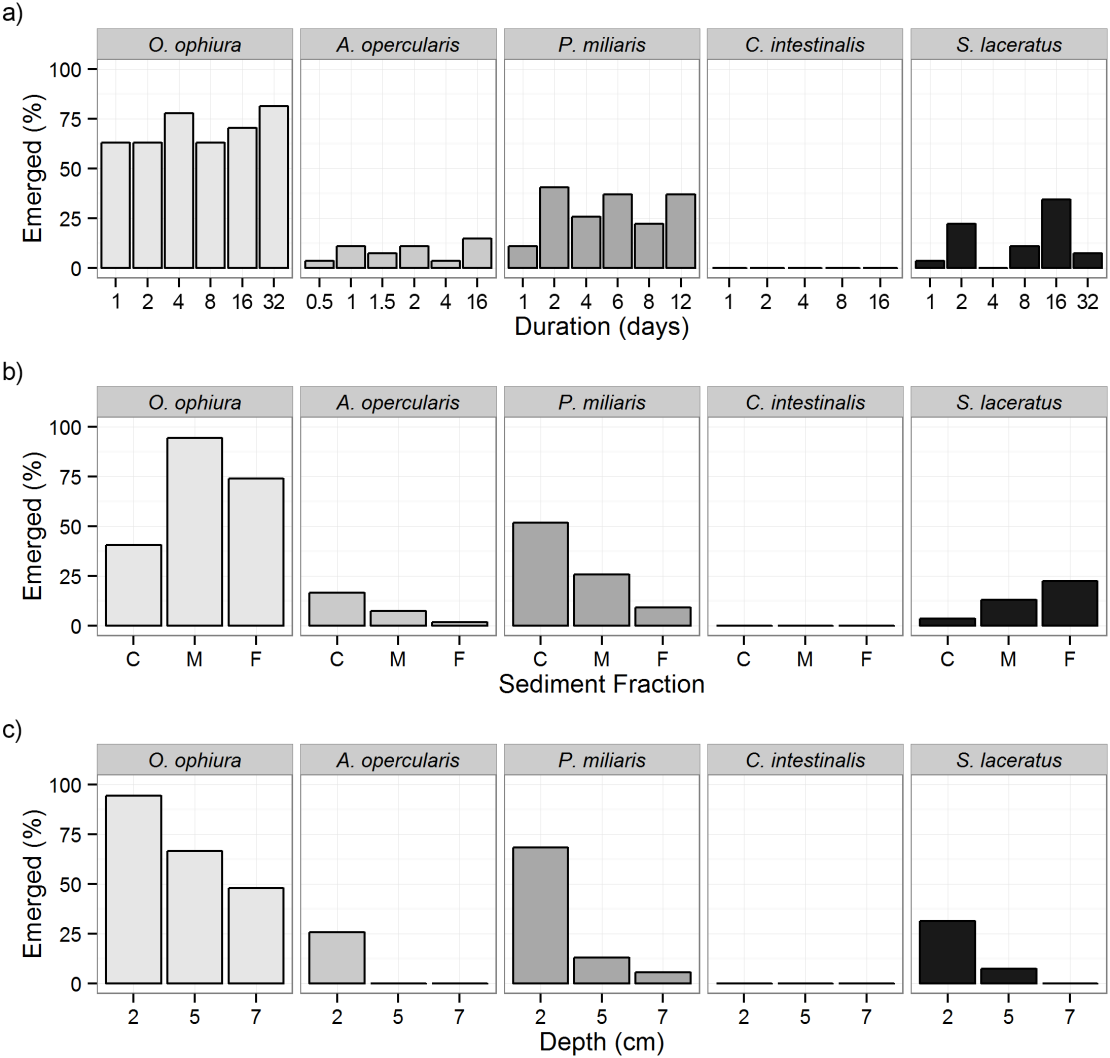
a-c. Multispecies assessment of emergence (%) in response to sediment burial. Factors assessed: a) Duration (days); b) Sediment fraction (C = course, M = medium, F = fine) and; c) Depth of burial (cm) above organism.

The results of the *O*. *ophiura* burial experiment demonstrate a high level of survivorship (9.9% overall mortality, n = 162). This is largely a reflection of the ability of the species to emerge from all depths of burial and all sediment fractions tested (69.8%), although statistical analysis confirmed that the depth of burial and the sediment fraction were both significant factors influencing the ability of *O*. *ophiura* to emerge (Table B in S1 File). Emergence from burial (Fig 3a) exceeded 60% in every duration of burial and was only marginally higher at 81.5% after 32 days of burial (n = 27 per duration). Emergence was highest under medium sediment (94.4%, n = 54, Fig 3b) and in shallow burials (94.4%, n = 54, Fig 3c), and lowest under coarse sediment (40.7%, n = 54), decreasing with increasing depth.

### Intolerance of *Sabellaria spinulosa* to burial

Mortality of buried *S*. *spinulosa* was 10% during the course of the experiment in comparison to 5% of control specimens (n = 60 and n = 40 respectively). The best fit minimal binomial glm indicated that burial was not a significant factor in *S*. *spinulosa* mortality, suggesting that these polychaetes are highly tolerant of burial (up to 32 days). Consequently no further statistical analysis of this data was under taken.

**Fig 4:**
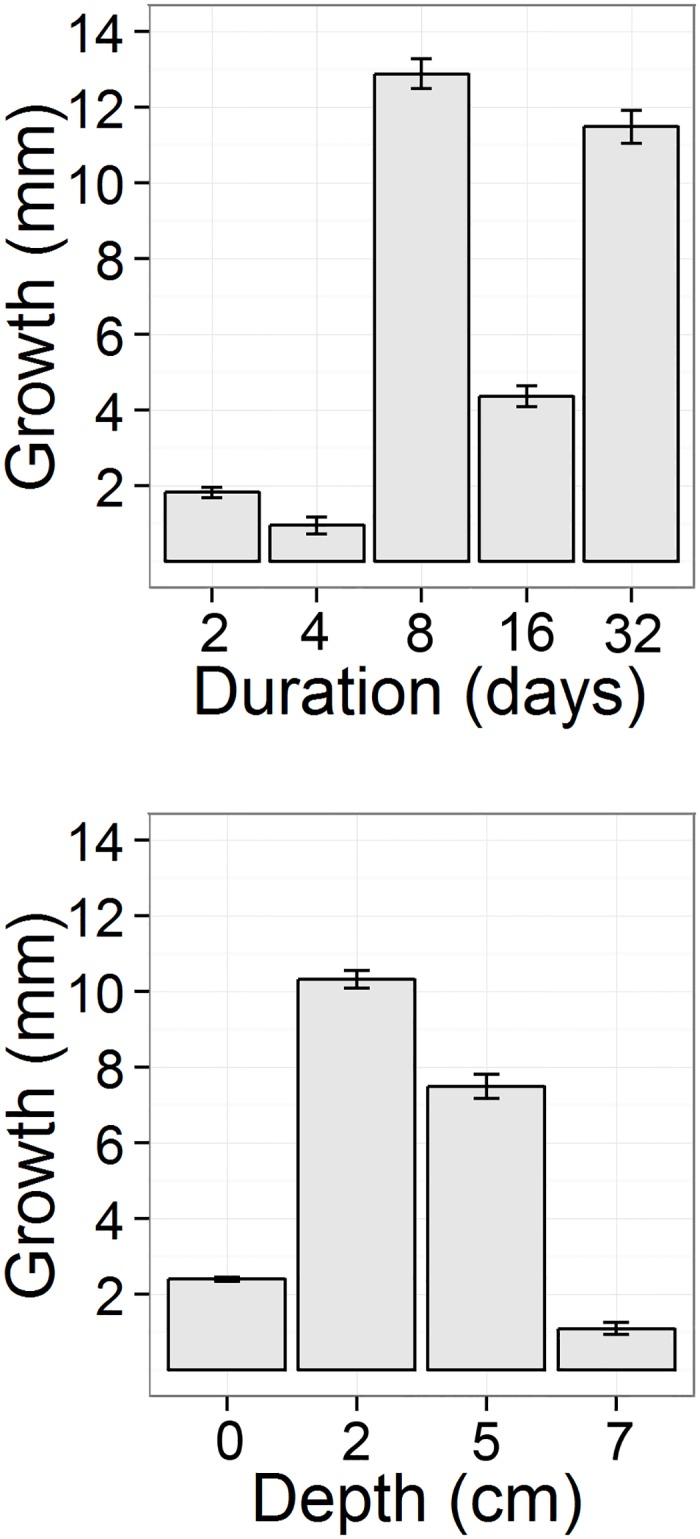
a-b. *Sabellaria spinulosa* assessment of dwelling tube growth (mm) in response to sediment burial. Factors assessed: a) Duration (days) and; b) Depth of burial (cm) above organism.

During the mortality assessment it was noted that under fine sediment some of the dwelling tubes had been extended during burial. In order to quantify this response, the fine sediment burial experiment was repeated as before. On this occasion, individual dwelling tubes were photographed and measured (using imageJ) before and after burial to assess the growth of dwelling tubes. The results of this trial are shown in Fig 4 where greatest dwelling tube growth was noted under shallow burial in medium sediment, and after ≥8 days.

### Intolerance of *Aequipecten opercularis* to burial

Size was not included in the bionomial glm for *A*. *opercularis* since all animals were a similar width ($\bar{\text{x}}$ = 53.84, sd = 1.93) and height ($\bar{\text{x}}$ = 52.29, sd = 1.97). The best fit minimal model indicated that the sediment fraction and an interaction between the duration and depth of burial were significant factors in *A*. *opecularis* mortality (Table C in S1 File). There was no mortality after 0.5 days but this increased to 29.6% after 1 day of burial and sharply increased to 74.1% and 88.9% after 2 and 4 days of burial respectively with similar levels of mortality after 16 days of burial (n = 27, Fig 2a). Mortality was similar under burials of coarse and medium sediment fractions (40.7 and 44.4% respectively) but more pronounced under fine sediment where 72.2% of individuals died (n = 54, Fig 2b). Mortality in *A*. *opercularis* was lowest under 2 cm (35%) of burial and higher under 5 and 7 cm of burial where 57.4% and 64.8% died, respectively (n = 54, Fig 2c). Survival of specimens that remained buried was low, with 100% mortality of individuals that remained buried after 32 days.

Survival was largely attributable to the ability to emerge from burial. The best fit minimal model which described the data for *A*. *opercularis* emergence indicated that the depth of burial and the sediment fraction were significant factors (Table D in S1 File). *A*. *opercularis* was most successful in emerging from coarse sediment (16.7%) and less successful in finer sediment fractions (Fig 3b). There was no emergence observed from medium (5 cm) or deep (7 cm) burials but 25.9% emerged from shallow (2 cm) burials (Fig 3c).

The results indicate that *A*. *opercularis* is highly intolerant of burial events, with the loss of over 70% of buried specimens after 2 days of burial (n = 27). Although the species demonstrated some ability to emerge from sediment this was limited in its extent (8.6%, n = 162), restricted to shallow (2 cm) burial and negatively influenced by finer sediment fractions.

### Intolerance of *Psammechinus miliaris* to burial

The best fit minimal model for *P*. *miliaris* indicated that sediment fraction and an interaction between the duration and depth of burial were significant factors for mortality (Table E in S1 File). There was no mortality in burials up to 4 days. After 6 days of burial however, 33.3% of *P*. *miliaris* had died and this increased to 44.4% after 8 days and 51.9% after 12 days (n = 27, Fig 2a). Mortality was minimal under coarse sediment but increased with progressively finer sediment to 44.4% mortality under fine sediment (n = 54, Fig 2b). Mortality was lowest under 2 cm of burial (14.8%) and similar under 5 and 7 cm of burial; 24.1 and 25.9% respectively (n = 54, Fig 2c).

Survival of *P*. *miliaris* was also largely due to the ability to emerge from burial. Statistical analysis indicated that significant factors influencing the emergence of *P*. *miliaris* were the depth of burial, the sediment fraction and most importantly, the size of the individual (Table F in S1 File) with larger individuals being less successful. The size of *P*. *miliaris* used in the study ranged from 19.0 mm to 40.2 mm ($\bar{\text{x}}$ = 28.3, sd = 4.14) and those capable of emerging were 19.0 to 34.6 mm ($\bar{\text{x}}$ = 26.2, sd = 3.91). The levels of emergence in *P*. *miliaris* did not increase after 2 days of burial (40.7%, n = 27, Fig 3a), and were much higher under coarse sediment (51.9%, n = 54) and shallow burials (68.5%, n = 54) although lower levels of emergence occurred in burials of all sediment fractions and all depths (Fig 3b and 3c).

Overall, 21.6% mortality (n = 162) of *P*. *miliaris* was observed. After the maximum of 12 days burial, mortality in animals that remained buried was high (82.4%, n = 17). Survivorship was partly due to the emergence (29.0%, n = 162) from burial which was most successful in coarse sediments and shallow burials.

### Intolerance of *Ciona intestinalis* to burial

Size of animals was not included in the statistical model for *C*. *intestinalis* since length could not be accurately assessed in this protean organism. An attempt was made at measuring mass but this was deemed inaccurate due the tunicates ability to take in or eject water.

The only significant factor influencing the mortality of *C*. *intestinalis* was found to be the duration of burial (Table G in S1 File). There was no mortality after 1 day of burial but after 2 days there was 100% mortality consistent in all burials of longer durations (Fig 2a). Mortality of *C*. *intestinalis* was not affected by sediment fraction or the depth of burial with mortality levels consistent at 80% at all depths and sediment fractions tested (Fig 2b and 2c).

The trial was terminated after 16 days since mortality was so high in shorter trials and emergence was never observed (Fig 3). It is apparent that *C*. *intestinalis* is highly intolerant of burial with 100% mortality of all individuals buried for at least two days (n = 108). Nevertheless, all specimens buried for only 24 hours survived (n = 27).

### Intolerance of *Sagartiogeton laceratus* to burial

Three anemones were inexplicably lost post-burial. These data points were omitted from the analysis which included one treatment and two control specimens.

The duration of burial was found to be the only significant factor influencing the mortality of *S*. *laceratus* (Table H in S1 File). There was no mortality in *S*. *laceratus* up to 16 days of burial however there was substantial mortality after 32 days where 48.1% died (n = 27, Fig 2a). Mortality was lowest under burial with coarse sediment and increased with progressively finer sediment to a maximum of 13.2% mortality under fine sediment burial (n = 53, Fig 2b). Mortality under varying depths of burial followed a similar trend although it was less pronounced with maximum mortality under the deep (7cm) burial where 11.3% died (n = 53, Fig 2c).

Emergence of *S*. *laceratus* was most significantly influenced by the depth of burial and the sediment fraction. An additional co-variable, the position of the burial chamber in the *p*VoRT, was also a significant factor in emergence from burial (Table I in S1 File) with emergence being more prevalent by individuals in burial chambers placed on the outside of the *p*VoRTs (76.2%, n = 21) compared to the inside. Emergence from burial was highest under the shallow burial (2 cm) where 31.5% individuals emerged (n = 53, Fig 3c). This was reduced under medium (5 cm, 14%, n = 54) burials and was not observed in the deep burials (7 cm, 0%, n = 54). In contrast to other species capable of emerging from burial, emergence was highest in the fine sediment burial where 22.6% of individuals emerged (n = 53) and lowest in coarse sediment (3.7%, n = 54, Fig 3b).

The results showed that *S*. *laceratus* is very tolerant of burial events, at least in the shorter term (≤ 16 days). The low overall mortality is largely a reflection of the species ability to survive under burial conditions, although the ability of the species to emerge also contributes to its survival.

## Discussion

The results of the burial experiments incorporating depth, duration and sediment fraction highlight large variation in survival and emergence response from burial in the species assessed. Although survival was generally highly dependent on depth and duration of burial as may be expected, emergence behaviour was not as predictable.

To facilitate a between species comparison we categorise burial intolerance as the proportion of animals that survive burial for 32 days. This categorization is similar to the MARLIN sensitivity assessment [23] but noteworthy is that the MarLIN burial depth is defined as 5 cm from the seabed, not, as is the case here, from the top of the animal. Our assessment revealed that *O*. *ophiura*, *A*. *opercularis* and *C*. *intestinalis* were all highly intolerant to burial whilst *P*. *miliaris* showed an intermediate intolerance and *S*. *laceratus* low intolerance. Certainly the most tolerant, with very high survival was *S*. *spinulosa*. With the exception of *C*. *intestinalis*, increasing duration and depth of burial with decreasing sediment fraction resulted in increased mortality for all species assessed. For *C*. *intestinalis* depth of burial and sediment fraction were found to be inconsequential since there was complete mortality of all specimens buried for more than one day.

When emergence from burial was assessed *O*. *ophiura* emerged most frequently, followed by *P*. *miliaris*. It is of note that whilst *O*. *ophiura* emerged most frequently from the medium and fine sediments, *P*. *miliaris* emerged more readily from coarse sediment. *A*. *opercularis* and *S*. *laceratus* showed similar emergence responses over time, with *A*. *opercularis* also demonstrating a higher frequency of emergence from coarse sediments whilst frequency of emergence of *S*. *laceratus* increased with progressively finer sediment. *C*. *intestinalis* specimens did not emerge irrespective of sediment fraction or depth of burial. Although some *S*. *spinulosa* ‘emergence tubes’ extended sufficiently to protrude beyond the sediment (as depicted in the inset in Fig 1), it was not possible to determine how many had reached the sediment-water interface and thus “emergence” was not quantified for this species. Finally, and perhaps unsurprisingly, the greatest ability to emerge from burial in all other species is from shallow (2 cm) burial.

Though not investigated as part of this study, two mytilids—the edible blue mussel (*Mytilus edulis*) and horse mussel (*Modiolus modiolus*) were assessed by Hutchison [26] using the same burial protocols as described here. Both species were capable of surviving sediment burial in the short term (<16 days) but with increasing burial duration mortality increased significantly, especially under finer sediment fractions. Whilst *M*. *modiolus* was more tolerant of short term burial, *M*. *edulis* was more tolerant of long term burial. The reason for this difference is that *M*. *edulis* was able to escape shallow (<2 cm) burial, a behavioural response absent in *M*. *modiolus*. Comparatively to the other species under test here, *M*. *edulis* can therefore be considered as having intermediate intolerance to burial whilst *M*. *modiolus* is highly intolerant.

Scallops and horse mussels are often found in close association [32] in mud, sand or gravelly habitats, usually with elevated SPM and often in tidally energetic areas [33]. *A*. *opercularis* was shown to be intolerant of sustained burial, unlike its more sedentary congener *Pecten maximus* [34], and this is probably due to its relatively high metabolism and oxygen demand [35]. *A*. *opercularis* is highly mobile and its escape response is to “swim” by rapid opening and closure of its shells. Unless a sediment deposition event is very sudden and relatively deep, it is likely to be able to avoid burial which is possibly why it has previously been ranked as tolerant to smothering [23]. Conversely, Hutchison [26] found that *M*. *modiolus*, which has no emergence response (at least in the laboratory), is highly intolerant to sustained burial which is somewhat surprising given that the species is often found partially buried [36]. However infaunal *Modiolus* beds tend to be in gravelly substrates which is in keeping with the results of Hutchison [26] who showed lower mortality in coarser sediment presumably since oxygenation of pore water would be higher [37]. Of interest is that scallop dredging, which is of considerable commercial importance [38], as well as other seabed fishing activities have led to the destruction of one of UKs largest *M*. *modiolus* reefs in Strangford Loch, Northern Ireland [30]. Although the physical impact of the trawl gear itself would have been the main contributing factor to reef loss, the deposition of sediments as a consequence of localised trawling [39] may have also contributed to reef decline. It should be noted though that other studies suggest sediment deposition from scallop trawling to be minimal [40].

The polychaete *S*. *spinulosa* is found exclusively in highly dynamic environments with elevated levels of suspended sediment considered essential in dwelling tube constructions [41]. Other than this study, no specific burial intolerance data was found for this species though the closely related polychaete *S*. *alveolata* was reported to survive short-term burial for days and even weeks in North Cornwall, Devon where sand depth may change by meters following storms [42]. Such supporting evidence together with the relatively low mortality of *S*. *spinulosa* recorded during this experiment, even following 32 days burial, has led to it being categorised as having a low intolerance to burial. Nevertheless, longer term burial of the sister species *S*. *alveolata* was suggested to be either detrimental to growth [43], or lethal [42], whilst excessive siltation has been shown to clog feeding apparatus [43].

The green urchin *P*. *miliaris* typically inhabits sheltered shores with boulders though can also be found on mixed coarse substrata or partially buried in course sand [23]. We originally hypothesised that due to its habitat preference and limited mobility it may be highly intolerant to burial. Nevertheless, our data show that *P*. *miliaris* is actually very adept at emerging from burial, second only to *O*. *ophiura* under test here. Indeed *P*. *miliaris* has been noted as the dominant species following dredging activities [44] and it has been suggested that areas with relatively impoverished epifauna due to strong near-bed tidal currents and high SPM, are often dominated by resilient motile species such as *P*. *miliaris* [45]. Like *A*. *opercularis* (and unlike the rest of the species assessed here), *P*. *miliaris* was better able to emerge from coarse sediment as opposed to fine; possibly a reflection of their adaptation to this type of habitat. Whilst *A*. *opercularis*, would rapidly open and close its shell to promote “swimming” shortly after being buried, the mechanism of emergence in *P*. *miliaris* is unknown. However, as with true burrowing echinoderms, it is likely that the large movable spines are used on the lateral portions of the ventral surface to propagate ditaxic waves front to rear, thereby allowing it to “dig” its way through the sediment [46]. It is suspected that this mechanism enabled some of the larger individuals in our trials to emerge from the deepest (7 cm) burial treatment.

Of the species under test here, the least tolerant to burial was *C*. *intestinalis* with total mortality after only two days and no emergence response. Tunicate respiration and feeding relies on regular water currents which are generated through the branchial basket, and their disruption, from stressors such as salinity shock or hypoxia, has rapid negative consequences to the condition of the animal [47]. Nevertheless, the frequency of occurrence of *C*. *intestinalis* in relatively turbid harbours suggests that this species is tolerant of elevated SPM and Naranjo [48] implied that the reason *C*. *intestinalis* has siphons with wide apertures is to reduce the risk of blockage. However from our data it is clear that the organisms do not tolerate direct burial and it is only by virtue of their habitat preference on rocks, boulders and especially man-made structures such as pier walls and ropes that they are unlikely to experience direct sediment burial.

As with *C*. *intestinalis*, the anemone *S*. *laceratus* preferentially attaches to hard substrates and can often be found vertically orientated. No prior details exist, to our knowledge, regarding its intolerance to sediment burial. Our findings show that it is relatively tolerant of burial which is somewhat surprising given its habitat preference. However, physiological studies on anemones suggests that they are capable of withstanding extended periods of hypoxia or at least, very reduced basal metabolism, when tentacles are retracted [49]. This coupled to their comparatively small size, relative to the other species, with resulting increased surface area ratio to interstitial pore water, may explain the high level of survival, even under deep burial. Of particular note was the ability of this species to emerge from burial, even from 5 cm depth. This emergence occurred by elongation of the animals “column” between the pedal disc and collar, substantially increasing the height of the animal and allowing its tentacles to reach the sediment/water interface. It seems likely that this is a behavioural adaptation that allows the animal to mitigate smothering, an interesting but possibly un-profound observation. Certainly some anemones actively bury/unbury themselves such as *Sagartia troglodytes* and *Cereus pedunculatus*, whilst a detailed study of *Phyllactis concinnata* revealed various types of behaviour associated with burial such as pharynx eversion and anti-peristaltic behaviour or crawling [50] which would greatly facilitate survival in such circumstances.

The results of the current study have enabled determination of the most basic factors contributing to mortality under burial and identified differing survival strategies between organisms. Although this study was facilitated by the use of *p*VoRT mesocosms that simulate water flow as representative of natural conditions, there are nevertheless factors in the field, not considered in this study, which will also influence mortality. For example, organic enrichment of natural sediments relative to those used in our study will reduce oxygen in pore water available to buried macrofauna. This has subsequently been quantified, at least for *M*. *edulis*, and shown to elevate mortality considerably [51]. When burial of habitats is gradual, as is often the case out-with the primary impact zone, the consequences of survival will undoubtedly be different than for sudden burial. Further the consequence of repeated burial and emergence/mortality following burial of large aggregations of individuals (for example mussel beds where integrity of structure is maintained by the connectivity of byssus threads between individual and the surrounding habitat) is currently unknown.

This study and those of Hutchison et al [26] and Cottrell et al [51], provide information that may aid the conservation of the experimental species and associated communities in the event of seabed disturbance due to anthropogenic activities. This is of particular importance where consent licenses for marine activities are to be granted and where the conservation importance of the affected habitat has been recognised as biogenic reefs of *S*. *spinulosa*, *M*. *edulis* or *M*. *modiolus* for example. It is therefore important that where anthropogenic sedimentation is expected to result in sudden burial, the species, particle size, depth of deposition and likely duration should be considered in conjunction with an assessment of the ecosystem “health” of the community. It is apparent that emergence from burial is a significant behavioural response in determining survival and one that not all benthic macroinvertebrate species possess—even species highly tolerant to burial would eventually die after prolonged periods under sediment without emergence. It was therefore of significance to find high levels of variation in individual escape ability as part of this study.

We conclude that responses to burial are highly variable between species and generalisations, as ever, are likely to be oversimplifications. It is possible, however, to make some species-specific predictions on burial intolerance and behavioural strategies for survival. These predictions may be used to inform environmental impact models that allow forecasting of the cumulative impacts of seabed disturbance and may provide mitigation measures for the sustainable use of the seabed.

## Supporting Information

S1 File **Table A. The summarised parameters of the minimal best fit binomial GLM, of mortality in *O*. *ophiura*.** Mortality is modelled as a function of the predictors: duration (log10), depth (2, 5 and 7 cm), sediment fraction (coarse, medium and fine), size of animal, and the interactions between the depth of burial with size of animal, and duration of burial with sediment fraction. **Table B. The summarised parameters of the minimal best fit binomial GLM of emergence in *O*. *ophiura*.** Emergence is modelled as a function of the predictors: depth (2, 5 and 7 cm) and sediment fraction (coarse, medium and fine). **Table C. The summarised parameters of the minimal best fit binomial GLM of mortality in *A*. *opercularis*.** Mortality is modelled as a function of the predictors: depth (2, 5 and 7 cm), sediment fraction (coarse, medium and fine) and the interaction between depth duration. **Table D. The summarised parameters of the minimal best fit binomial GLM of emergence in *A*. *opercularis*.** Emergence is modelled as a function of the predictors: depth (2, 5 and 7 cm) and sediment fraction (coarse, medium and fine). **Table E. The summarised parameters of the minimal best fit binomial GLM of mortality in *P*. *miliaris*.** Mortality is modelled as a function of the predictors: duration (log10), depth (2, 5 and 7cm), sediment fraction (coarse, medium and fine), and interactions of duration with depth. **Table F. The summarised parameters of the minimal best fit binomial GLM of emergence in *P*. *miliaris*.** Emergence is modelled as a function of the predictors: depth (2, 5 and 7 cm), sediment fraction (coarse, medium and fine) and size. **Table G. The summarised parameters of the minimal best fit binomial GLM of emergence in *C*. *intestinalis*.** Emergence is modelled as a function of the predictor: duration (log10). **Table H. The summarised parameters of the minimal best fit binomial GLM of emergence in *S*. *lacerates*.** Emergence is modelled as a function of the predictor: duration (log10). **Table I. The summarised parameters of the minimal best fit binomial GLM of emergence in *S*. *lacerates*.** Emergence is modelled as a function of the predictors: depth (2, 5 and 7 cm), sediment fraction (coarse, medium and fine) and position in the *p*VoRTs.
(DOCX)
