# Supplementary material for: Sediment Burial Intolerance of Marine Macroinvertebrates
Source: PLoS One. 2016 Feb 22;11(2):e0149114. doi: 10.1371/journal.pone.0149114 (PMC4765823; doi:10.1371/journal.pone.0149114)
Supplement: S1 File — Table A. The summarised parameters of the minimal best fit binomial GLM, of mortality in O. ophiura. Mortality is modelled as a function of the predictors: duration (log10), depth (2, 5 and 7 cm), sediment fraction (coarse, medium and fine), size of animal, and the interactions between the depth of burial with size of animal, and duration of burial with sediment fraction. Table B. The summarised parameters of the minimal best fit binomial GLM of emergence in O. ophiura. Emergence is modelled as a function of the predictors: depth (2, 5 and 7 cm) and sediment fraction (coarse, medium and fine). Table C. The summarised parameters of the minimal best fit binomial GLM of mortality in A. opercularis. Mortality is modelled as a function of the predictors: depth (2, 5 and 7 cm), sediment fraction (coarse, medium and fine) and the interaction between depth duration. Table D. The summarised parameters of the minimal best fit binomial GLM of emergence in A. opercularis. Emergence is modelled as a function of the predictors: depth (2, 5 and 7 cm) and sediment fraction (coarse, medium and fine). Table E. The summarised parameters of the minimal best fit binomial GLM of mortality in P. miliaris. Mortality is modelled as a function of the predictors: duration (log10), depth (2, 5 and 7cm), sediment fraction (coarse, medium and fine), and interactions of duration with depth. Table F. The summarised parameters of the minimal best fit binomial GLM of emergence in P. miliaris. Emergence is modelled as a function of the predictors: depth (2, 5 and 7 cm), sediment fraction (coarse, medium and fine) and size. Table G. The summarised parameters of the minimal best fit binomial GLM of emergence in C. intestinalis. Emergence is modelled as a function of the predictor: duration (log10). Table H. The summarised parameters of the minimal best fit binomial GLM of emergence in S. lacerates. Emergence is modelled as a function of the predictor: duration (log10). Table I. The summarised parameter [file pone.0149114.s001.docx]

**S1 Table A:**

|  | **Estimate** | **Std. Error** | **Z value** | **Pr(>\|z\|)** |
| --- | --- | --- | --- | --- |
| (Intercept) | -11.49 | 4.93 | -2.330 | **0.0198** |
| (Log10) Duration | 4.29 | 2.19 | 1.961 | **0.0499** |
| Depth 2 cm | -15.49 | 14518.29 | -0.001 | 0.9991 |
| Depth 5cm | -92.14 | 66.31 | -1.390 | 0.1646 |
| Sediment Fine | 2.75 | 2.58 | 1.064 | 0.2872 |
| Sediment Medium | 4.52 | 2.79 | 1.623 | 0.1045 |
| Size | 0.87 | 0.49 | 1.778 | 0.0753 |
| Depth (2 cm):Size | -0.64 | 2097.84 | 0.000 | 0.9998 |
| Depth (5cm):Size | 11.81 | 8.43 | 1.400 | 0.1616 |
| (log10)Duration:Sediment Fine | -1.40 | 2.35 | -0.595 | 0.5516 |
| (log10)Duration:Sediment Medium | -9.57 | 4.52 | -2.117 | **0.0343** |
| Null deviance: | 93.83 on 153 df | | | |
| Residual deviance: | 37.83 on 143 df | | | |
| AIC: | 59.83 | | | |
| Number of Fisher Scoring iterations: | 19 | | | |

**S1 Table B:**

|  | **Estimate** | **Std. Error** | **Z value** | **Pr(>\|z\|)** |
| --- | --- | --- | --- | --- |
| (Intercept) | -2.26 | 0.56 | -4.011 | **6.04e-05** |
| Depth 2cm | 4.04 | 0.80 | 5.049 | **4.43e-07** |
| Depth 5cm | 1.28 | 0.54 | 2.389 | **0.016898** |
| Sediment Fine | 2.16 | 0.56 | 3.858 | **0.000114** |
| Sediment Medium | 4.20 | 0.78 | 5.412 | **6.25e-08** |
| Null deviance: | 198.59 on 161 df | | | |
| Residual deviance: | 115.29 on 157 df | | | |
| AIC: | 125.29 | | | |
| Number of Fisher Scoring iterations: | 6 | | | |

**S1 Table C:**

|  | **Estimate** | **Std. Error** | **Z value** | **Pr(>\|z\|)** |
| --- | --- | --- | --- | --- |
| (Intercept) | -2.93 | 0.71 | -4.120 | **3.80e-05** |
| (log10) Duration | 2.88 | 0.85 | 3.392 | **0.000694** |
| Depth 5cm | 0.57 | 0.75 | 0.755 | 0.450395 |
| Depth 7cm | 0.80 | 0.83 | 0.967 | 0.333502 |
| Sediment Fine | 2.81 | 0.68 | 4.115 | **3.87e-05** |
| Sediment Medium | 0.36 | 0.61 | 0.602 | 0.547460 |
| (log10) Duration:Depth 5cm | 4.99 | 2.23 | 2.237 | **0.025288** |
| (log10)Duration:Depth 7cm | 9.17 | 3.51 | 2.611 | **0.009030** |
| Null deviance: | 224.18 on 161 df | | | |
| Residual deviance: | 105.40 on 154 df | | | |
| AIC: | 121.4 | | | |
| Number of Fisher Scoring iterations: | 7 | | | |

**S1 Table D:**

|  | **Estimate** | **Std. Error** | **Z value** | **Pr(>\|z\|)** |
| --- | --- | --- | --- | --- |
| (Intercept) | -2.601e-16 | 4.714e-01 | 0.000 | 1.0000 |
| Depth 5cm | -1.964e+01 | 2.270e+03 | -0.009 | 0.9931 |
| Depth 7cm | -1.964e+01 | 2.270e+03 | -0.009 | 0.9931 |
| Sediment Fine | -2.833e+00 | 1.132e+00 | -2.503 | **0.0123** |
| Sediment Medium | -1.253e+00 | 7.373e-01 | -1.699 | 0.0893 |
| Null deviance: | 95.31 on 161 df | | | |
| Residual deviance: | 51.75 on 156 df | | | |
| AIC: | 61.75 | | | |
| Number of Fisher Scoring iterations: | 19 | | | |

**S1 Table E:**

|  | **Estimate** | **Std. Error** | **Z value** | **Pr(>\|z\|)** |
| --- | --- | --- | --- | --- |
| (Intercept) | -20.20 | 5.38 | -3.753 | **0.000175** |
| (log10) Duration | 16.87 | 5.26 | 3.208 | **0.001334** |
| Depth 7cm | -0.27 | 5.23 | -0.051 | 0.959146 |
| Depth 5cm | 7.57 | 4.72 | 1.604 | 0.108715 |
| Sediment Fine | 7.96 | 1.82 | 4.386 | **1.16e-05** |
| Sediment Medium | 4.64 | 1.51 | 3.076 | **0.002095** |
| (log10)Duration:Depth 7cm | 0.76 | 6.00 | 0.127 | 0.899205 |
| (log10)Duration:Depth 5cm | -10.86 | 5.61 | -1.935 | 0.053023 |
| Null deviance: | 169.08 on 161 df | | | |
| Residual deviance: | 55.45 on 154 df | | | |
| AIC: | 71.45 | | | |
| Number of Fisher Scoring iterations: | 8 | | | |

**S1 Table F:** The summarised parameters of the minimal best fit binomial GLM of emergence in *P. miliaris* as a function of the predictors depth (2, 5 and 7 cm), sediment fraction (coarse, medium and fine) and size.

|  | **Estimate** | **Std. Error** | **Z value** | **Pr(>\|z\|)** |
| --- | --- | --- | --- | --- |
| (Intercept) | 4.19 | 2.19 | 1.912 | 0.0558 |
| Depth 7cm | -1.39 | 0.83 | -1.684 | 0.0922 |
| Depth 5cm | 20.85 | 1674.50 | 0.012 | 0.9901 |
| Sediment Fine | -21.69 | 1674.50 | -0.013 | 0.9897 |
| Sediment Medium | -18.85 | 1674.50 | -0.011 | 0.9910 |
| Size | -0.17 | 0.08 | -2.159 | **0.0308** |
| Null deviance: | 195.13 on 161 df | | | |
| Residual deviance: | 75.08 on 156 df | | | |
| AIC: | 87.08 | | | |
| Number of Fisher Scoring iterations: | 19 | | | |

**S1 Table G:** The summarised parameters of the minimal best fit binomial GLM of emergence in *C. intestinalis* as a function of the predictor duration (log10).

|  | **Estimate** | **Std. Error** | **Z value** | **Pr(>\|z\|)** |
| --- | --- | --- | --- | --- |
| (Intercept) | -23.77 | 16916.78 | -0.001 | 0.999 |
| (Log10)Duration | 156.50 | 72264.44 | 0.002 | 0.998 |
| Null deviance: | 1.3511e+02 on 134 df | | | |
| Residual deviance: | 6.5044e-09 on 133 df | | | |
| AIC: | 4 | | | |
| Number of Fisher Scoring iterations: | 25 | | | |

**S1 Table H:**

|  | **Estimate** | **Std. Error** | **Z value** | **Pr(>\|z\|)** |
| --- | --- | --- | --- | --- |
| (Intercept) | -99.99 | 13476.18 | -0.007 | 0.994 |
| (log10)Duration | 66.38 | 8953.38 | 0.007 | 0.994 |
| Null deviance: | 90.35 on 160 df | | | |
| Residual deviance: | 37.39 on 159 df | | | |
| AIC: | 41.39 | | | |
| Number of Fisher Scoring iterations: | 22 | | | |

**S1 Table I:**

|  | **Estimate** | **Std. Error** | **Z value** | **Pr(>\|z\|)** |
| --- | --- | --- | --- | --- |
| (Intercept) | -3.178 | 0.88 | -3.599 | **0.00032** |
| Depth 5 cm | -1.89 | 0.64 | -2.927 | **0.00343** |
| cDepth 7 cm | -18.95 | 1364.69 | -0.014 | 0.98892 |
| Sediment Fine | 2.39 | 0.87 | 2.740 | **0.00614** |
| Sediment Medium | 1.47 | 0.89 | 1.652 | 0.09852 |
| Position (outside) | 1.56 | 0.62 | 2.543 | **0.01098** |
| Null deviance: | 124.68 on 160 df | | | |
| Residual deviance: | 77.84 on 155 df | | | |
| AIC: | 89.84 | | | |
| Number of Fisher Scoring iterations: | 18 | | | |
